# The prevalence of *Yersinia enterocolitica* in game animals in Poland

**DOI:** 10.1371/journal.pone.0195136

**Published:** 2018-03-29

**Authors:** Kinga Syczyło, Aleksandra Platt-Samoraj, Agata Bancerz-Kisiel, Anna Szczerba-Turek, Joanna Pajdak-Czaus, Sebastian Łabuć, Zbigniew Procajło, Piotr Socha, Gulzhan Chuzhebayeva, Wojciech Szweda

**Affiliations:** 1 Department of Epizootiology, Faculty of Veterinary Medicine, University of Warmia and Mazury in Olsztyn, Oczapowskiego 13, Olsztyn, Poland; 2 Department of Animal Reproduction with Clinic, Faculty of Veterinary Medicine, University of Warmia and Mazury in Olsztyn, Oczapowskiego 13, Olsztyn, Poland; 3 Department of Veterinary Public Health, Faculty of Veterinary and Livestock Technology, Baitursynov Kostanay State University, Baitursynov 47, Kostanay, Kazakhstan; Defense Threat Reduction Agency, UNITED STATES

## Abstract

Natural reservoirs of *Yersinia (Y*.*) enterocolitica* comprise different animal species, but little is known about the role of wild animals in the epidemiology of yersiniosis. The aim of the study was to evaluate the prevalence of *Y*. *enterocolitica* among game animals in Poland. The bio-serotypes and the pathogenicity markers of the analyzed isolates were determined. The experimental material comprised rectal swabs from 857 free-living animals hunter-harvested over a period of 2 years (2013–2014) in hunting districts across Poland. The isolates from bacteriological studies were confirmed by PCR and bio-serotyped based on the results of biochemical and agglutination tests. In the group of the 218 analyzed isolates of *Y*. *enterocolitica*, 133 were derived from wild boars, 70 from red deer, 11 from roe deer and 4 from fallow deer, and they accounted for 61.0%, 32.1%, 5.1% and 1.8% of all isolates, respectively. Bio-serotyping assays revealed that 91.7% of the examined isolates belonged to biotype 1A (200/218). The remaining 18 isolates belonged to bio-serotypes 1B/NI (3/218, 1.4%), 1B/O:8 (1/218, 0.5%), 2/NI (6/218, 2.8%), 2/O:27 (1/218, 0.5%), 2/O:3 (1/218, 0.5%), 2/O:9 (2/218, 0.9%), 3/NI (2/218, 0.9%), 4/O:3 (1/218, 0.5%) and 4/O:9 (1/218, 0.5%). The *ail* gene, a suggestive virulence gene for *Y*. *enterocolitica*, has been found in 30 isolates from 20 wild boars, in 6 isolates from red deer, and in 1 isolate from roe deer. Our study demonstrated that *Y*. *enterocolitica* is frequently isolated from game animals in Poland, which poses a risk of spreading these infectious agents to other animal species and humans.

## Introduction

*Yersinia (Y)*. *enterocolitica*, a member of the *Enterobacteriaceae* family, is the etiological agent of yersiniosis, an important zoonotic disease that causes symptoms ranging from a mild, self-limiting diarrhea to acute mesenteric lymphadenitis, and can sometimes develop into a variety of parenteral forms [1–3]. In Europe, yersiniosis is a notifiable zoonotic disease which must be reported to authorities and presented in the annual report of the European Food Safety Authority (EFSA). For several years, *Y*. *enterocolitica* has ranked third among the pathogens that most frequently cause gastrointestinal disorders in Europe, after *Campylobacter* spp. and *Salmonella* spp. In 2015, 7202 cases of yersiniosis were recorded in Europe [4].

*Y*. *enterocolitica* is relatively resistant to external factors, and as a psychrotolerant pathogens, it can survive in the environment for a long time. *Y*. *enterocolitica* has been divided into more than 70 serotypes based on differences in the structure of the somatic antigen, and into 6 biotypes based on its biochemical characteristics. Most of *Y*. *enterocolitica* strains that are pathogenic to humans belong to biotypes 1B 2–5, whereas biotype 1A (BT1A) is regarded as nonpathogenic [1,2,5,6]. However, selected strains of this biotype are considered as opportunistic [7,8,9].

The pathogenicity of *Y*. *enterocolitica* is related to the presence of chromosomal and plasmid genes which control the production and functions of proteins that facilitate survival in the host body and are responsible for the clinical signs of disease [10–12]. There is a strong relationship between pathogenicity and the presence of chromosomal *ail* and *ystA* genes in *Y*. *enterocolitica* [10,12]. The BT1A isolates are usually *ail* and *ystA* negative, and they may harbor *ystB* or *ystC* genes that control the production of heat-stable enterotoxins [8,11]. The presence of *ystB* has been reported in approximately 80% of *Y*. *enterocolitica* BT1A isolates [8].

The epidemiology of yersiniosis is complex and not fully understood. The pig is the main reservoir of human-pathogenic strains, most of which cause asymptomatic infections, colonize tonsils and the digestive tract, and are excreted with feces. Infected animals become long-term carriers [1,13]. Different species of farm, domestic, free-living and game animals can also act as natural reservoirs of *Y*. *enterocolitica*. Despite the scant availability of information on the occurrence of *Y*. *enterocolitica* in game animals, these hosts seem to be an important reservoir of *Y*. *enterocolitica*, and they contribute to the environmental circulation of the bacterium [14–17]. Epidemiological links between free-living animals vs. farm and domestic animals have not been identified to date [17].

Poland has a temperate climate with average annual temperature of 7 ^0^C and relatively cold winters, which enables psychrotolerant bacteria such as *Yersinia* spp. to survive in the environment [18]. Poland has a long tradition of hunting, and game meat is widely consumed. Game animals of different species make up a substantial part of wildlife in Polish forest ecosystems. Selected species, such as wild boars and free-living ruminants, travel many miles in search of food or for reproductive purposes. Animals that are carriers of human pathogens can contribute to the transmission of pathogens across considerable distances. This study investigates the transmission of *Y*. *enterocolitica* by game animals, and it can shed new light on the epidemiology of infections and reduce the prevalence of yersiniosis in humans. This is the first large-scale study into the dissemination of *Y*. *enterocolitica* in game animals in Poland.

The purpose of the study was to determine the prevalence of *Y*. *enterocolitica* infections among game animals in Poland with the use of traditional culturing and molecular methods. The isolates were divided into different biotype, serotype and virulence marker groups, to explore the significance of game animals as a reservoir and a vector in the spread of *Y*. *enterocolitica* infections.

## Materials and methods

The investigated material comprised rectal swabs from 857 game animals: wild boars (*Sus scrofa*) (n = 434), red deer (*Cervus elaphus*) (n = 291), roe deer (*Capreolus capreolus*), (n = 117) and fallow deer (*Dama dama*) (n = 15), hunted in 12 out of 16 Polish regions during the hunting seasons of 2013–2014. The animals were hunted by authorized hunters under a grant from the Polish National Centre for Research and Development. The health status of the animals was not recorded. Samples for analysis were collected in duplicate from each animal before evisceration, they were immediately placed in tubes with the Amies transport medium and delivered to the laboratory within 48 h. A total of 1714 samples were analyzed.

The animals were not hunter-harvested exclusively for the needs of this study. All samples were collected during routine examinations of game meat, therefore, ethical approval for animal experimentation was not required.

### Bacteriological analysis

For each duplicate sample, one rectal swab was analyzed by adding 9 ml of ITC warm enrichment broth (irgasan, ticarcillin, potassium chlorate) and incubating at 25 ^0^C for 48 h, and the other sample was placed in 9 ml of cold enrichment PSB broth (peptone, sorbitol, bile salts) and incubated at 4 ^0^C for 3 weeks to determine its ability to grow at a low temperature. Warm and cold enrichments were tested in accordance with PN-EN ISO 10273 [19]. After incubation, 0.5 ml of each enrichment was immersed in 4.5 ml of 0.5% KOH in 0.5% NaCl for 20 s to reduce background microflora. It was then streaked onto the CIN medium (Yersinia Selective Agar, DIFCO) and incubated at 30 ^0^C for 48 h. in an aerobic atmosphere. Isolates with a growth pattern indicative of *Y*. *enterocolitica* on CIN (small and smooth, with the a red center and a translucent rim), which were motile at 22 ^0^C, but not at 37 ^0^C were stored on agar slants at 4 ^0^C for biotyping, serotyping and molecular analyses. Additionally, biochemical identification was performed using the API 20E bacterial identification system (bioMerieux) according to the manufacturer’s instructions.

### DNA isolation

Bacterial DNA was extracted with the Genomic Mini kit (Biotechnology A&A, Poland) according to the manufacturer’s procedures. The extracted nucleic acid was stored at -20 ^0^C until further analyses.

### Multiplex PCR

The identity of *Y*. *enterocolitica* isolates was confirmed and their pathogenic characteristics were determined by amplifying *ail*, *yst A* and *ystB* genes as markers of virulence. Primer sequences are specified in S1 Table. The primers were synthesized in the DNA Sequencing Laboratory of the Polish Academy of Sciences, Oligo, Warsaw, Poland. The HotStartTaq Plus DNA Polymerase Kit (Qiagen) and the HotStartTaq *Plus* Master Mix Kit (Qiagen) were used in the multiplex PCR technique. The reaction was performed according to the following schedule: final concentration of MgCl2−1.5 nM, initial denaturation at 94 ^0^C for 120 s, followed by 30 cycles of DNA amplification: denaturation at 94 ^0^C for 90 s, annealing at 46 ^0^C for 60 s, polymerization at 72 ^0^C for 120 s, and final polymerization at 72 ^0^C for 5 min.

The products were separated by electrophoresis in 2% agarose gel with the Midori Green Advanced DNA Stain (Nippon Genetics Europe GmbH, Germany) in 1x TAE buffer. The products were visualized under UV light. PCR results were analyzed and archived with the use of the GelDoc gel documentation system (Bio-Rad).

The specificity of the reaction was confirmed by sequencing amplicons (Genomed, Poland). The analyzed nucleotide sequences are available in the GenBank database under accession numbers [KJ592623-KJ592627], [KM253258-KM253268] and [KT209961-KT209996].

### Biotype identification

Biotypes were identified according to the method proposed by Wauters [5,6] and described in the PN-EN ISO 10273 [19]. PCR-confirmed *Y*. *enterocolitica* isolates were examined for their ability to produce salicin acid, trehalose and xylose, and for esculin fermentation. They were also evaluated for the presence of pyrazinamidase and indole as well as the ability to reduce nitrogen compounds. The results of the applied biochemical tests are interpreted in S2 Table.

### Serotype identification

The serotype of *Y*. *enterocolitica* isolates was determined by the slide agglutination test with the use of commercial sera for antigens O:3, O:5, O:8, O:9 (ITEST, Hradec Kralove, Czech Republic) and O:27 (SEFIN, Berlin, Germany). Live bacterial cells cultured for 24 hours on blood agar (Grasco) were used as the antigen. Bacterial cells were suspended in a drop of 0.85% NaCl, placed on slides and combined with a drop of each of the sera. Agglutination after 1 min of shaking in an orbital shaker was regarded as a positive result. In the absence of agglutination with any of the available diagnostic sera, the isolate was marked as NI (non-identified).

### Statistical analysis

The results were processed statistically with the use of a descriptive statistics module, correlation analysis and a non-parametric statistics module. Calculations were performed in STATISTICA PL v.12 software. The Chi-squared non parametric test with Yates’ correction [20] was used. The calculations were performed at a significance level α = 0.05.

## Results

**Fig 1:**
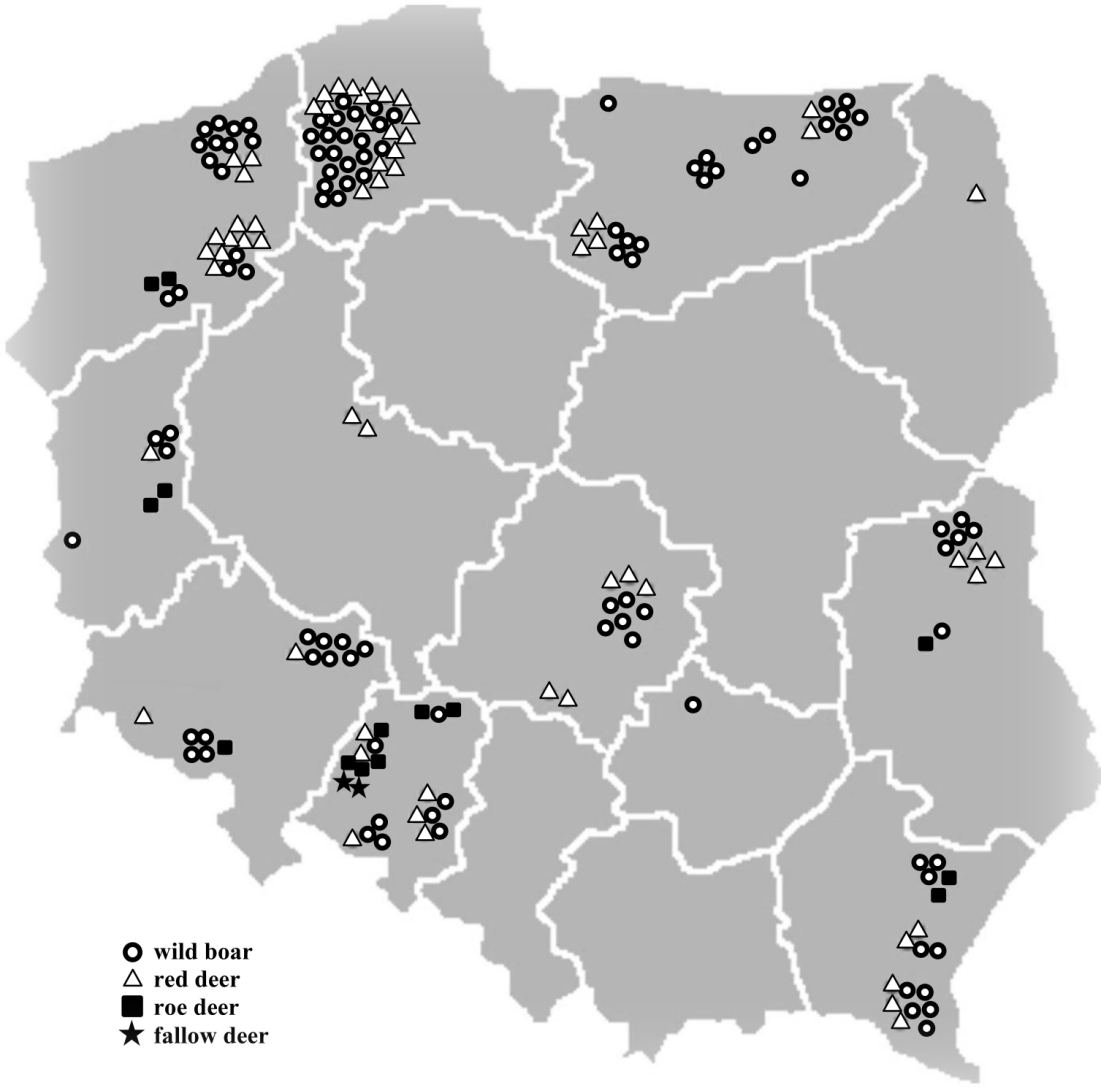
Geographical distribution of *Yersinia enterocolitica* positive samples in the Polish regions.

*Y*. *enterocolitica* isolates were detected in the rectal swabs of the 21.7% (186/857) of the tested animals. The results of culture tests and molecular analyses are shown in Table 1, and the geographical origin of the positive samples is presented in Fig 1.

**Table 1:** Results of the bacteriological study, including the number of *Yersinia* spp. confirmed by PCR.

Species	Examined animals	*Y*. *enterocolitica-*positive animals (%)	*Y*. *enterocolitica* isolates confirmed by PCR		
			ITC	PSB	total no. of isolates
Wild boar	434	110 (25.3%)	49	84	133
Red deer	291	63 (21.6%)	27	43	70
Roe deer	117	11 (9.4%)	3	8	11
Fallow deer	15	2 (13,3%)	2	2	4
**Total**	**857**	**186 (21.7%)**	**81**	**137**	**218**

**ITC–***Yersinia* isolates from warm enrichment; **PSB—***Yersinia* isolates from cold enrichment

The prevalence of *Y*. *enterocolitica* infections (110/434) was highest in wild boars, where 25.3% of the examined animals were infected. In comparison, only 21.6% red deer (63/291), 9.4% roe deer (11/117) and 13.3% fallow deer (2/15) were infected. Highly significant (p<0.01) differences were confirmed in the frequency of the isolation of *Y*. *enterocolitica* isolates from wild boar and red deer versus roe deer and fallow deer.

The biotype and serotype characteristics of *Y*. *enterocolitica* isolates are shown in Table 2. Bioserotyping tests demonstrated that 91.7% of the isolates belonged to BT1A (200/218), serotypes O:27 (n = 4), O:3 (n = 7), O:5 (n = 10), O:8 (n = 10) and O:9 (n = 9). Most isolates of BT1A (160/200) did not agglutinate with the applied diagnostic sera, and they were determined as NI (non-identified). The remaining 18 isolates belonged to the bio-serotypes 1B/NI (3/218, 1.4%), 1B/O:8 (1/218, 0.5%), 2/NI (6/218, 2.8%), 2/O:27 (1/218, 0.5%), 2/O:3 (1/218, 0.5%), 2/O:9 (2/218, 0.9%), 3/NI (2/218, 0.9%), 4/O:3 (1/218, 0.5%) and 4/O:9 (1/218, 0.5%).

**Table 2:** Biotype and serotype characteristics of *Yersinia enterocolitica* isolates.

Biotype/serotype	Number of *Yersinia enterocolitica* isolates number			
	Wild boar	Red deer	Roe deer	Fallow deer
1A/NI	107	46	5	2
1A/O:27	3	1		
1A/O:3	1	6		
1A/O:5	2	4	3	1
1A/O:8	7	3		
1A/O:9	3	3	2	1
1B/NI	3			
1B/O:8	1			
2/NI	2	3	1	
2/O:27		1		
2/O:3	1			
2/O:9	2			
3/NI	1	1		
4/O:3		1		
4/O:9		1		
**TOTAL**	**133**	**70**	**11**	**4**

NI–non-identified serotypes

Most of the 218 analyzed *Y*. *enterocolitica* isolates (n = 137, 62.8%) were obtained from cold enrichment. In 36 cases (wild boar n = 24, red deer n = 10, fallow deer n = 2), isolates were obtained from both warm and cold enrichments, and in this group, biotype/serotype differences were noted in 14 cases. In 8 wild boars isolates were obtained simultaneously from warm and cold enrichments and they were classified into the following biotypes/serotypes: 2/NI and 1A/NI, 1A/O:27 and 1A/NI, 1B/NI and 1A/NI, 2/NI and 1A/NI, 1A/NI and 1A/O:27, 1B/O:8 and 2/O:9, 1A/NI and 1A/O:8, 1A/NI and 2/NI. The isolates from 4 red deer were classified as: 1A/O:8 and 1A/NI, 1A/NI and 2/NI, 1A/O:5 and 1A/O:3, 1A/NI and BT2/NI. The isolates from 2 fallow deer were classified as: 1A/O:9 and 1A/NI, 1A/O:5 and 1A/NI.

The prevalence of *ail*, *ystA* and *ystB* genes in *Y*. *enterocolitica* isolates is presented in Tables 3 and 4. The *ail* gene, which is highly suggestive for pathogenic *Y*. *enterocolitica* isolates, was found in 30 isolates from 20 wild boars and it appeared simultaneously with the *ystA* gene in only 2 cases. In the remaining 28 isolates, the *ail* gene occurred simultaneously with the *ystB* gene, and 21 of these isolates belonged to BT1A.

**Table 3:** The prevalence of *ail*, *ystA* and *ystB* genes in *Yersinia enterocolitica* isolates from game animals.

Animal species	Prevalence of genes		
	*ail*	*ystA*	*ystB*
Wild boar	30	2	131
Red deer	6	1	70
Roe deer	1	1	10
Fallow deer	0	0	4
***Total***	**37**	**4**	**215**

**Table 4:** The prevalence of *ail*, *ystA* and *ystB* genes in *Yersinia enterocolitica* biotype 1B, 2–4 isolates from game animals.

NO	ANIMAL SPECIES	BIOTYPE	SEROTYPE	GENES
1	**Wild boar**	2	O:9	*ail*, *ystB*
2		2	NI	*ail*, *ystB*
3		2	O:3	*ail*, *ystA*
4		1B	NI	*ystB*
5		1B	NI	*ystB*
6		1B	NI	*ystB*
7		3	NI	*ystB*
8		2	NI	*ystB*
9		1B	O:8	*ystB*
10		2	O:9	*ystB*
11	**Red deer**	4	O:3	*ail*, *ystA*
12		3	NI	*ystB*
13		2	O:27	*ystB*
14		4	O:9	*ail*, *ystA*
15		2	NI	*ystB*
16		2	NI	*ystB*
17		2	NI	*ystB*
18	**Roe deer**	2	NI	*ystB*

NI- non-identified serotypes

The *ail* gene was found in 6/70 *Y*. *enterocolitica* isolates from red deer. Only in one case, the *ail* gene occurred together with the *ystA* gene. In the remaining 5 cases, the *ail* gene was present with the *ystB* gene. Most of *Y*. *enterocolitica* isolates from red deer harbored only the *ystB* gene. In roe deer, the *ail* gene was found in 1 out of 11 isolates, and it was accompanied by the *ystB* gene. Single *ystB* genes were found in 11 isolates.

The *ail* gene was not detected in samples from fallow deer, and all 4 *Y*. *enterocolitica* isolates from this animal species contained the *ystB* gene. In our study, 37 out 218 (17.0%) isolates contained the *ail* gene, but 21 of BT1A isolates with the *ail* gene also harbored *ystB*. Detailed molecular characteristics and the results of a phylogenetic analysis of isolates harboring *ail* and *ystB* were described in a previous paper [21].

*Y*. *enterocolitica* isolates were detected in rectal swabs from all examined animal species, and they were most prevalent in wild boars. Most isolates belonged to the BT1A/NI group and harbored the *ystB* gene.

## Discussion

The present study makes the first ever attempt to identify the prevalence of *Y*. *enterocolitica* in wild animals based on samples collected from animals that were hunter-harvested in all controlled hunting zones in Poland. The pathogen was isolated from 21.7% of 857 examined animals, which clearly indicates that this bacterium is widespread among game animals in Poland. Most controlled hunting zones in Poland are situated in large forests which occupy vast parts of the country, except central Poland. *Y*. *enterocolitica* was isolated in each hunting district where test samples were collected (Fig 1).

Free-living animals seem to play an important role in the epidemiology of yersiniosis. It should be noted that the consumption of game meat increases the risk of foodborne diseases [14,17,22]. There is a general scarcity of published data on the prevalence of foodborne infections caused by the consumption of game meat contaminated with *Y*. *enterocolitica*. However, the pathogen can contaminate the carcasses of infected game animals when the intestines are damaged by shot pellets or during evisceration. The contamination of game meat with *Y*. *enterocolitica* also remains insufficiently investigated. Bucher et al. [23] detected pathogenic *Y*. *enterocolitica* on the surface of 38.8% raw game meat samples in Bavaria, Germany. Avagnina et al. [24] isolated *Y*. *enterocolitica* from 2.4% examined carcasses of large game animals in Italy. In Poland, Bancerz-Kisiel et al. [25] detected *Y*. *enterocolitica* isolates in cold stored carcasses in 60% of the examined roe deer, 43.8% of red deer and 55% of wild boars.

Relatively little is known about the occurrence of *Y*. *enterocolitica* infections in wild animals in the world. According to some studies, wild boars are a significant reservoir of strains pathogenic to humans [14,22,26–28]. Serological investigations in northeastern Germany and Switzerland demonstrated the presence of antibodies against *Yersinia* spp. in the sera of more than 60% of the examined wild boars [22,29]. Bacteriological analyses conducted by von Altrock [28] revealed the presence of *Y*. *enterocolitica* in the tonsils of 17.1% of the examined wild boars in Lower Saxony, Germany.

Our study demonstrated that other species of game animals, including red deer, roe deer and fallow deer, can also be a source of infections of *Y*. *enterocolitica*. The highest number of positive samples was found in wild boars (25.3%), which is consistent with the results of the cited studies. This result could be partly attributed to the high genetic affinity of wild boars and pigs. Pigs are the natural hosts and reservoirs of the discussed pathogen. However, significant percentages of *Y*. *enterocolitica* isolates also obtained from red deer, roe deer and fallow deer (21.7%, 9.4% and 13.3%, respectively) in our study (Table 1). These findings provide evidence that wild boars are not the only species of game animals that play a role in dissemination of *Y*. *enterocolitica*.

Most isolates were classified as BT1A (91.7%). Similar results were observed by other researchers [17,27,28] and in our previous study into *Y*. *enterocolitica* in wild animals [14,26,30–32]. However, we also detected 18 isolates (0.8%) belonging to biotypes 2, 3, 4 and 1B (Tables 2 and 4). In Europe, the most common pathogenic bio-serotypes isolated in clinical cases of yersiniosis are serotypes O:9 and O:5.27 of BT2, serotypes O:1, 2, 3, O:5.27 of BT3, serotype O:3 of BT4 and serotypes O:2, 3 of BT5 [1]. In our study, only 6 out of 18 isolates of BT2 (O:9, O:27, O:3), BT4 (O:3, O:9) and 1B (O:8) could be considered as virulent on the basis of bio-serotype typing (Table 2). The pathogenic characteristics of 3 isolates were confirmed by molecular analysis which revealed the presence of *ail* and *ystA* genes (Table 4).

In 2014, 244 cases of yersiniosis were recorded in Poland. The incidence rate was 0.63/100,000 inhabitants, and most infections were caused by *Y*. *enterocolitica* bio-serotypes 4/O:3 (88%), 1B/O:8 (6.9%), and 2/O:9 (5.2%) [33]. The clinical significance of *Y*. *enterocolitica* BT1A is difficult to determine, mainly due to the lack of pYV plasmids which are the most important markers of the pathogenic *Y*. *enterocolitica*. Until recently, BT1A strains were defined as non-pathogenic. In recent years, they have been increasingly isolated from clinical cases of yersiniosis, although in some cases, the symptoms of yersiniosis were not specific and could be caused by another pathogen [7–9]. However, there is clinical evidence that selected BT1A strains of *Y*. *enterocolitica* can cause gastrointestinal symptoms [8]. Little is known about the pathogenic mechanisms of disease caused by *Y*. *enterocolitica* BT1A. This study revealed that the YST-b enterotoxin encoded by the *ystB* gene in most BT1A isolates was the major contributor to diarrhea caused by *Y*. *enterocolitica* of this biotype [7,8,11]

The identification of one 1B/O:8 *Y*. *enterocolitica* isolate in our study is noteworthy. This bio-serotype is rare, and it is isolated mainly in North America. The first highly pathogenic 1B/O:8 strain of *Y*. *enterocolitica* was isolated in Europe in 2003. In Poland, the prevalence of yersiniosis has increased gradually since 2004, and in 2008 O:8 strains of *Y*. *enterocolitica* outnumbered O:3 and O:9 serotypes which has been predominant in previous years [34]. The reservoir of *Y*. *enterocolitica* 1B/O:8 in Poland has not yet been identified [34]. However, our isolate harbored only the *ystB* gene (Table 4).

The EFSA has recommended biotyping as the simplest method of evaluating the pathogenicity of *Y*. *enterocolitica* [4]. Our experience suggests that determination of the pathogenicity by biotyping/serotyping is a time-consuming and laborious process. In some cases, the results were ambiguous and required replication. Virulence markers were quickly and reliably identified by PCR, but the results were difficult to interpret when *ail* and *ystB* were detected simultaneously. Therefore, both methods should be used to *Y*. *enterocolitica* in wildlife specimens.

The isolation and characterization of *Y*. *enterocolitica* require some technical effort because the growth of this microorganism could be inhibited by the antagonistic effects of background flora [35]. The method applied in the present study is useful for isolating *Y*. *enterocolitica* from environmental samples [32]. The high frequency (186/857, 21.7%) of *Y*. *enterocolitica* detections in game animals can be attributed to the combined application of both methods, and most of the isolates in the present study originated from cold enrichment (137/218, 62.8%).

In this study, virulence markers *ail*, *ystA* and *ystB* were amplified in a molecular analysis to determine the pathogenicity of isolates. More importantly, this procedure was applied to confirm and verify the results of the bacteriological methods. The amplification of the *ail* gene is particularly useful for determining the virulence properties of *Y*. *enterocolitica* [2,10,36,37].

In conclusion, most *Y*. *enterocolitica* isolates from game animals belonged to the BT1A, but enteropathogenic *Y*. *enterocolitica* bio-serotypes which are common in European pigs were also identified in hunted game animals. Our results confirm the high prevalence of *Y*. *enterocolitica* in Poland and indicate that game animals, especially wild boars, are important vectors of infections. Red deer appear to be more susceptible to *Y*. *enterocolitica* infections than other wild ruminants. The prevalence of the identified virulence factors and the bio-serotype affiliation of the isolates from game animals inhabiting different Polish regions clearly indicate that *Y*. *enterocolitica* is widely disseminated in Poland.

## Supporting information

S1 Table Primer sequences for amplifying the *ail*, *yst A* and *yst B* genes. (DOCX)

S2 Table Biochemical tests used for biotyping *Yersinia enterocolitica* isolates. (DOCX)
