# Supplementary material for: The prevalence of Yersinia enterocolitica in game animals in Poland
Source: PLoS One. 2018 Mar 29;13(3):e0195136. doi: 10.1371/journal.pone.0195136 (PMC5875811; doi:10.1371/journal.pone.0195136)
Supplement: S1 Table — (DOCX) [file pone.0195136.s001.docx]

###### S1 Table. Primer sequences for amplifying the *ail, yst A* and *yst B* genes.

| **Gene** | **Primer sequences** | **Product size (bp – base pairs)** | **Source** |
| --- | --- | --- | --- |
| *ail* | 5’TGGTTATGCGCAAAGCCATGT3’  5’TGGAAGTGGGTTGAATTGCA 3’ | 356 | [10] |
| *ystA* | 5’GTCTTCATTTGGAGGATTCGGC3’  5’AATCACTACTGACTTCGGCTGG3’ | 134 | [10] |
| *ystB* | 5’TGTCAGCATTTATTCTCAACT3’  5’GCCGATAATGTATCATCAAG3’ | 180 | [37] |
