# Supplementary material for: The prevalence of Yersinia enterocolitica in game animals in Poland
Source: PLoS One. 2018 Mar 29;13(3):e0195136. doi: 10.1371/journal.pone.0195136 (PMC5875811; doi:10.1371/journal.pone.0195136)
Supplement: S2 Table — (DOCX) [file pone.0195136.s002.docx]

**S2 Table. Biochemical tests used for biotyping *Yersinia enterocolitica* isolates**

| TEST | BIOTYPE | | | | | |
| --- | --- | --- | --- | --- | --- | --- |
|  | 1A | 1B | 2 | 3 | 4 | 5 |
| Salicin acid production in 24 h | + | − | − | − | − | − |
| Trehaloze production | + | + | + | + | + | − |
| Xyloze production | + | + | + | + | − | v |
| Esculin fermentation in 24 h | +/− | − | − | − | − | − |
| Pyrazinamidaze presence | + | − | − | − | − | − |
| Indole production | + | + | v | − | − | − |
| Nitrate reduction | + | + | + | + | + | − |

v - variable
